# Developmental potential of human oocytes matured in vitro followed by vitrification and activation

**DOI:** 10.1186/1757-2215-6-30

**Published:** 2013-04-18

**Authors:** Patrick Imesch, David Scheiner, Min Xie, Daniel Fink, Erwin Macas, Raghvendra Dubey, Bruno Imthurn

**Affiliations:** 1 Department of Gynecology, University Hospital Zurich, Frauenklinikstrasse 10, Zurich, CH-8091, Switzerland; 2 Division of Reproductive Endocrinology, University Hospital Zurich, Zurich, CH-8091, Switzerland

**Keywords:** In vitro maturation, Fertility preservation, Vitrification, Ovarian tissue, Parthenogenesis

## Abstract

**Background:**

Oocyte in vitro maturation (IVM) and cryopreservation at the time of routine ovarian tissue freezing may be offered to cancer patients as an additional option for fertility preservation. This study aimed to investigate the developmental capacity of oocytes isolated from unstimulated ovaries.

**Methods:**

Immature oocytes (*n* = 63) from seven consenting premenopausal patients were analysed. Oocytes were collected during routine laparoscopic examination with biopsy of an ovary (cystic adnexal mass, *n* = 3; cervical adenocarcinoma, *n* = 2) or oophorectomy (sex reassignment surgery, *n* = 2) without previous stimulation of the ovaries. The stage of the patient’s menstrual cycle was not considered. Oocytes in all visible antral follicles were aspirated from ovaries, cultured in IVM medium and vitrified at the MII stage before being kept in liquid nitrogen for at least one month. After warming, oocytes were subjected to parthenogenetic activation by chemical stimulus. Their further development was recorded at intervals of 24 hours for up to 6 days of culture.

**Results:**

61.9% of oocytes matured *in vitro* within 48 hours. The survival rate after vitrification and warming was 61.5%. A total of 75% of surviving oocytes were able to respond to artificial activation, 44.4% of the parthenotes developed to early embryonic stage. However, only 1 in 18 (5.6%) of the resulting embryos reached blastocyst stage.

**Conclusions:**

Oocytes matured *in vitro* from unstimulated ovaries seem to have limited developmental potential after cryopreservation and artificial activation. Although the outcome of IVM for non-stimulated oocytes is poor, it is currently the only chance besides cryopreservation of ovarian tissue for women for whom ovarian stimulation is not possible due to life circumstances. Based on our preliminary results, we suggest that the use of cryopreserved ovaries for fertility preservation in women with cancer warrants further investigation.

## Background

Fertility preservation in women with cancer is increasingly in demand due to improvements in diagnosis, effective treatment and follow-up, leading to better patient survival, especially among younger women [1,2]. The deleterious effects on ovaries of aggressive chemo- and radio-therapy are well known. Depending on treatment regimen, individual circumstances and response to treatment, some patients recover and can lead a normal, fertile life, while others may suffer permanent loss of gonadal function and become sterile [3-5]. Thus, the preservation of reproductive potential in women prior to treatment for cancer is becoming increasingly important. Recently, awareness of fertility preservation has also been raised in women with non-oncological conditions such as haematological and auto-immunological diseases that require similar treatments as for cancer. Such treatments can also cause premature ovarian failure or even total loss of fertility [6].

Currently, one method used to potentially preserve a woman’s fertility is to freeze ovarian cortical tissue before the patient undergoes radio- and/or chemotherapy. However, depending on the type and stage of cancer—especially blood-borne cancer and those with potential danger of metastasis to ovaries—there is a risk of transmission of malignant cells when ovarian cortical tissue is cryopreserved and later used to restore fertility by auto-grafting (retransplantation) [7-9]. In the latter situation, isolation of oocytes from antral follicles at the time of freezing of the ovarian tissue, with maturation *in vitro* before the oocytes are cryopreserved, can be considered as an alternative approach. However, little is known about the developmental capacity of such oocytes following cryopreservation and fertilization. This study therefore aimed to investigate the developmental potential of oocytes matured *in vitro* after cryopreservation and artificial activation by chemical stimulus.

## Methods

Between October 2010 and January 2012, seven patients (age ranged from 18 to 41 years) were recruited at the Department of Gynecology, University Hospital of Zurich. Participation was voluntary, and written informed consent was obtained before the procedure. Inclusion criteria for patient recruitment were a pre-menopausal status and the feasibility of oocyte retrieval: all recruited patients were scheduled to undergo a laparoscopic procedure. Medical indications for laparoscopy were female-to-male sex reassignment surgery (SRS) (*n* = 2), benign cystic adnexal mass (*n* = 3), and cervical adenocarcinoma (*n* = 2). In the case of SRS, bilateral oopherectomy was performed, while in the other cases an ovarian biopsy was taken. Due to the required medical therapy, the stage of menstrual cycle at the time of oocyte collection could not be taken into account and was therefore ignored. In addition, no ovarian stimulation (FSH or HCG priming) was performed in order to avoid further physical burden to the patients. The two patients who underwent SRS had preoperative hormonal treatment with androgens.

### Oocyte retrieval

Oocytes were retrieved by biopsy of the ovary. Ex vivo, visible (size not measured) follicles were aspirated under reduced pressure of 85 mm Hg using a 19-gauge single-lumen aspiration needle (K-OPS-7035-RWH-ET; Cook, Australia), according to the transvaginal aspiration technique described by Chian *et al*. [10]. The aspirated fluid was collected in 10 ml culture tubes containing 2 ml pre-warmed 0.9% saline solution with 2 IU/ml heparin. For bilateral oophorectomy, ovaries were transported immediately in IVM (*in vitro* maturation) medium containing HEPES (Sage; CooperSurgical, USA) to the IVF laboratory (on the same floor of the hospital). Antral follicles visible on the surface of ovaries were aspirated using a 1 ml syringe fitted with a 19-gauge needle and flushed with IVM medium. The collected fluid was examined for oocyte-cumulus-complex under a stereomicroscope, and maturity was determined under an inverted microscope.

### In vitro maturation and vitrification

The immature oocytes were cultured in IVM medium (Sage; CooperSurgical, USA) supplemented with 75 mIU/ml FSH and 75 mIU/ml LH at 37°C in an atmosphere of 6% CO_2_ in air with high humidity for up to 48 hours. About 24 hours after the incubation, the oocyte-cumulus-complex was denuded with 80 IU/ml hyaluronidase solution (Vitrolife, Sweden) in order to assess maturity. Mature oocytes (MII) were vitrified immediately using a Kitazato Vitrification Kit (Japan). CryoTop was used as the carrier. Any immature oocytes were cultured for an additional 24 hours. Vitrification was repeated for any further mature oocytes observed.

### Oocyte activation and development

Oocytes were kept in liquid nitrogen for at least one month before being warmed (Kitazato Warming Kit). Thawed oocytes were allowed to recover for a minimum of 1 hour at 37°C in a 6% CO_2_ incubator before being subjected to artificial activation (parthenogenesis). Only viable cells were subjected to chemical activation after warming. For ethical reasons, oocyte activation using chemical stimulus as described by Polak de Fried *et al*. [11] was conducted to simulate the process of fertilization. In brief, oocytes were incubated with 10 μM ionomycin (Sigma) in G-Mops medium supplemented with 5% HSA (Vitrolife, Sweden) at 37°C in room air for 6 minutes before placing in culture medium containing 2 mM 6-dimethylamimopurine (6-DMAP, Sigma) for 3 hours at 37°C / 6% CO_2_. The oocytes were analysed for successful activation 16–20 hours later by assessing the presence of a single pronucleus within the cytoplasm without a second polar body. Further development was recorded at intervals of 24 hours for up to 6 days of culture.

Statistical evaluation was undertaken using Intercooled Stata 10.0 (StataCorp LP, College Station, TX) by means of Fisher’s exact test for categorical data. P-values below 0.05 indicate statistical significance.

The study was reviewed and approved by the Ethics Committee of Canton Zurich (KEK-ZH-NR: 2010-0169/0).

## Results

Table 1 shows the basic characteristics of the oocytes from the seven patients and summarises the results. From the seven patients, a total of 63 viable immature oocytes was obtained, with numbers ranging from 4 to 19 oocytes per patient. At the time of collection, all oocytes were at the germinal vesicle (GV) stage. In total, 61.9% (39/63) of immature oocytes (GV) reached stage MII following maturation for up to 48 hours in vitro, with a rate of 35.9% (14/39) IVM in the first 24 hours. The survival rate after vitrification and warming was 61.5% (24/39). 75% (18/24) of the surviving oocytes were able to respond to artificial activation, and 44.4% (8/18) of them developed to the 4- to 6-cell stage (blastomere). Only one (1/18, 5.6%) of the resulting embryos (parthenotes) reached the blastocyst stage. Analyzing for different patient diagnosis, no statistically significant differences (P=0.269) were found in regard to oocyte development (Table 2).

**Table 1:** Outcomes for oocytes collected from ovaries

**Patient**	**Age**^**a**^	**No. viable oocytes collected**	**IVM to MII for up to 48 h (%)**^**b**^	**No. oocytes survived after warming (%)**	**No. oocytes activated (%)**	**No. oocytes further developed (up to 6 days) (%)**	**Patient diagnosis**
1	18	9	2	1	1	0	Sex reassignment surgery
2	24	19	14	10	7	4^c^	Sex reassignment surgery
3	25	11	9	4	3	0	Cystic adnexal mass
4	29	4	2	2	2	2	Cystic adnexal mass
5	39	5	2	2	1	1	Cystic adnexal mass
6	37	5	5	4	3	0	Cervical adenocarcinoma
7	41	10	5	1	1	1	Cervical adenocarcinoma
Total		63	39/63 (61.9%)	24/39 (61.5%)	18/24 (75%)	8/18 (44.4%)	8/63 (12.7%)
P value^d^			0.84	0.53	1.0	0.70	

No statistically significant differences were found between different patient diagnosis (SRS, adnexal mass, and adenocarcinoma) at each step (IVM, oocyte activation and development).

^a^ Age of patient at the time of oocyte collection.

^b^ All MII oocytes were vitrified after IVM.

^c^ One embryo reached the blastocyst stage.

^d^ Fisher’s exact test.

IVM, in vitro maturation; MII, metaphase II.

**Table 2:** Development of collected oocytes after IVM, vitrification, and parthenogenetic activation according to patient diagnosis

**Patient diagnosis**	**No. oocytes collected**	**No. oocytes further developed (up to 6 days)**	**Success (%)**
Sex reassignment surgery	28	4	14.3%
Cystic adnexal mass	20	3	15%
Cervical adenocarcinoma	15	1	6.7%
P value ^a^			0.269

No statistically significant differences were found for further development of oocytes between the different patient diagnoses.

^a^ Fisher’s exact test.

## Discussion

Our results show that IVM can be achieved in immature oocytes isolated from ovaries without taking account of the phase of the menstrual cycle in which they are collected, and without any hormonal stimulation. There are some reports on IVM and cryopreservation of immature oocytes retrieved during routine ovarian tissue cryobanking for female patients with cancer, suggesting the possibility of combined fertility preservation [12-17]. Revel *et al*. were the first to demonstrate that, without any hormonal stimulation, oocytes retrieved from ovarian tissue dissection can be matured *in vitro* and cryopreserved, or fertilized by ICSI followed by freezing as an additional option for fertility preservation [12]. However, to our knowledge, no report has shown any further investigation of the fertilization and developmental potential of such oocytes after IVM and cryopreservation.

In our study, we achieved an IVM rate of 61.9%. A study by Fasano et al. [15] reported a rate of 31% only, using the same commercial IVM medium and same concentrations of gonadotrophin supplements. After vitrification and warming, 75% of the surviving oocytes in our study responded to activation, which is higher than the expected rate of 67% according to the findings of Paffoni *et al*. [18]. Together with the survival rate of 61.5% after vitrification and warming, our results indicate the development potential of oocytes matured *in vitro* followed by cryopreservation and activation. However, more than half of the activated oocytes in our study arrested at the pronucleus stage. Nearly all of the dividing parthenotes failed to develop beyond the 6-cell stage, and only one embryo reached blastocyst stage. The higher IVM rate in our study may be explained by the small cohort of seven patients representing a less oncologic collective, as some oncologic patients are found to exhibit impaired potential of oocyte development, although this remains controversial [19].

Using fresh, mature oocytes donated by patients from controlled ovarian hyperstimulation cycles, Paffoni *et al*. have shown that parthenogenetic activation using ionomycin and 6-DMAP as chemical stimuli is as effective as fertilization by ICSI, with 67.3% after activation and 62.3% after fertilization [18]. But these authors also observed that the rate of developmental arrest was significantly higher among activated oocytes (32.9%) than among fertilized oocytes (8.5%), and the blastocyst rate was 12.8%. Although Polak de Fried *et al*. have also reported an activation rate of 86.1% using noninseminated, cryopreserved human oocytes under similar conditions, the blastocyst rate was 16.7% [11]. Based on these results, we cannot exclude the possibility that insufficiency of artificial activation might affect embryo development, causing early developmental arrest.

The size of dominant follicles at the time of oocyte collection determines the outcome of *in vitro* maturation and embryo development to blastocyst stage [20]. HCG priming 36 hours before oocyte retrieval increases the rate of IVM and embryo development significantly, resulting in many live births [21,22]. Since the criteria of patient recruitment were based mainly on their age (pre-menopausal) and the feasibility of additional oocyte retrieval, i.e., that the required surgical procedure would not be compromised, the stage of the menstrual cycle at the time of oocyte collection was not considered. This in part reflects the real clinical situation when dealing with immediate ovarian tissue cryopreservation for cancer patients. Thus, while this could have contributed to the very low rate of blastocyst formation in our results, our procedure of retrieving oocytes irrespective of menstrual cycle stage or medical preconditioning reflects the actual clinical situation for cryopreservation of ovarian tissue for women with cancer.

The development of oocyte cryopreservation has made rapid progress since the introduction of vitrification techniques, and rates of fertilization, development and implantation have improved significantly, with survival rates of more than 85% and pregnancy rates of over 40% [23-25]. It has been further demonstrated that vitrification is superior to conventional slow freezing procedures in terms of meiotic spindle maintenance and recovery during and after the freezing process [26-28]. Therefore, in this study we used vitrification to cryopreserve oocytes matured *in vitro*. The relatively low survival rate of 61.5% could reflect oocyte quality before vitrification. Since the above-cited results by others were obtained from either oocyte donation programs or patients seeking fertility treatment, the oocytes were likely to be healthier than those from the present cohort of diseased patients in this study. In clinical practice, however, patients requiring fertility preservation due to cancer-related diseases are often in a most vulnerable state, as reflected in our study and also in a recent report by Escriba et al. [17]. The latter authors found that 36.1% of the immature oocytes isolated during routine ovarian tissue cryopreservation for oncology patients achieved spontaneous nuclear maturation in vitro. However, only 41% of them responded to parthenogenetic activation. It can be speculated therefore that the outcome of IVM and oocyte cryopreservation in such cases is less promising than would be predicted by the results of IVM used for infertility treatment.

The limitation of our study is the small number of patients and collected oocytes in this heterogenous patient collective. However, with the exception of the SRS group, this might in part reflect the real clinical situation when dealing with immediate ovarian tissue cryopreservation for cancer patients. We cannot ignore the fact that pretreatment with androgens for SRS patients might also have had a negative influence on oocyte quality, although the only blastocyst was generated from one of the two SRS cases. In addition, no statistically significant differences were found for the SRS group compared with the other subject groups in our study. Another limitation in the current study is that, for ethical reasons, we are not allowed to perform studies using fertilization. Using artificial activation only we were not able to reveal the true developmental potential of oocytes matured in vitro. An improvement on the study design would be to include a control group. In theory, a control could consist of immature oocytes from patients undergoing hormonal stimulation for IVF. Unfortunately, our daily routine has shown that these by-products are often in a state of degeneration, thus limiting their use as a control.

## Conclusions

This first attempt suggests that oocytes matured *in vitro* from unstimulated ovaries have limited developmental potential following cryopreservation and artificial activation. However, although the outcome of IVM for non-stimulated oocytes is poor, this method is currently the only chance besides cryopreservation of ovarian tissue for women for whom time-consuming hormonal stimulation is not possible due to life circumstances, e.g. cancer. We hope that our findings will provide a useful stimulus for further investigation aimed at modifying and optimizing the methodology to achieve successful *in vitro* culture and development of oocytes isolated from ovaries during routine ovarian tissue cryopreservation, particularly from patients with malignant disease.

## Abbreviations

FSH: Follicle-stimulating hormone; GV: Germinal vesicle; HCG: Human chorionic gonadotropin; ICSI: Intracytoplasmic sperm injection; IVF: In vitro fertilization; IVM: In vitro maturation; MII: Metaphase II; SRS: Female-to-male sex reassignment surgery.

## Competing interests

The authors declare that they have no competing interests.

## Authors’ contributions

MX, EM, RD, and BI conceived and designed the experiments. MX and EM performed the experiments. MX, PI, DS, DF, RD, and BI analyzed the data. PI, DS, and DF contributed reagents/materials/analysis tools. PI, DS, and MX wrote the paper. All authors read and approved the final manuscript.
